# The Yin and Yang of the Bone Marrow Microenvironment: Pros and Cons of Mesenchymal Stromal Cells in Acute Myeloid Leukemia

**DOI:** 10.3389/fonc.2019.01135

**Published:** 2019-10-25

**Authors:** Marilena Ciciarello, Giulia Corradi, Federica Loscocco, Giuseppe Visani, Federica Monaco, Michele Cavo, Antonio Curti, Alessandro Isidori

**Affiliations:** ^1^Department of Experimental, Diagnostic and Specialty Medicine, Institute of Hematology “L. & A. Seràgnoli”, University of Bologna, S. Orsola-Malpighi Hospital, Bologna, Italy; ^2^Hematology and Stem Cell Transplant Center, AORMN Hospital, Pesaro, Italy; ^3^Department of Hematology and Oncology, Institute of Hematology “L. and A. Seràgnoli”, University Hospital S.Orsola-Malpighi, Bologna, Italy

**Keywords:** acute myeloid leukemia, mesenchymal stromal cells, bone marrow microenvironment, drug resistance, immunomodulation

## Abstract

Mesenchymal stromal cells (MSCs) have, for a long time, been recognized as pivotal contributors in the set up and maintenance of the hematopoietic stem cell (HSC) niche, as well as in the development and differentiation of the lympho-hematopoietic system. MSCs also have a unique immunomodulatory capacity, which makes them able to affect, both *in vitro* and *in vivo*, the function of immune cells. These features, namely the facilitation of stem cell engraftment and the inhibition of lymphocyte responses, have both proven essential for successful allogeneic stem cell transplantation (allo-SCT), which remains the only curative option for several hematologic malignancies. For example, in steroid-refractory acute graft-vs. host disease developing after allo-SCT, MSCs have produced significant results and are now considered a treatment option. However, more recently, the other side of the MSC coin has been unveiled, because of their emerging role in creating a protective and immune-tolerant microenvironment able to support the survival of leukemic cells and affect the response to therapies. In this light, it has been proposed that the failure of current treatments to efficiently override the stroma-mediated protection of leukemic cells accounts for the high rate of relapse in acute myeloid leukemia, at least in part. In this review, we will focus on emerging microenvironment-driven mechanisms conferring a survival advantage to leukemic cells overt physiological HSCs. This body of evidence increasingly highlights the opportunity to consider tumor-microenvironment interactions when designing new therapeutic strategies.

## Introduction

A “Mesenchymal stem cell-like” population was initially classified as a subpopulation of bone marrow (BM) cells with the ability to reconstitute ectopic BM following heterotopic transplantation (1, 2). This subpopulation showed rapid adherence to culture dishes and a fibroblast-like shape, which distinguished them from hematopoietic cells. In addition, this subpopulation retained the ability to form colonies, called colony-forming unit fibroblasts (CFU-Fs) (3), with the capacity to regenerate bone tissue in serial implants *in vivo*, suggesting a self-renewal and multi-lineage potential (4). Subsequent studies identified the role of the adherent cells obtained from long-term BM cultures in supporting hematopoietic cells (5). The hypothesis of a *stem cell niche*, with non-hematopoietic components regulating hematopoiesis, was born (6), although it took several more years to definitively accept this concept. In 1991, Caplan coined the definition of *Mesenchymal Stem Cells* for this adherent cell population (7). Nowadays, the term of Mesenchymal Stromal Cells (MSCs) seems more appropriate Indeed, the multi-lineage potential of a single MSC has not been fully proven, thus not all MSCs can be considered *bona fide* stem cells *in vivo* (8).

Since the first definition, the studies regarding MSC properties have advanced rapidly. Thus, beside the originally discovered function in hematopoiesis supporting ability, other talents have been revealed *in vitro* and *in vivo*. The physiological abilities of MSCs, as we will discuss below (YIN), have proven to be essentials for successful allogeneic stem cell transplantation in AML and to counteract the Graft versus host disease (GVHD) (YIN), but can also be exploited by malignant cells to their advantage (YANG). The BM microenvironment can be remodeled favoring malignant cell expansion and resistance to therapy, at the expense of normal hematopoiesis. Thus, the “Pros” of MSCs may become “Cons” in the malignant niche.

## The Yin of MSCs: MSC Role in Healthy Hematopoiesis and Immune Modulation

### MSC-Dependent Hematopoiesis Support

**Figure 1:**
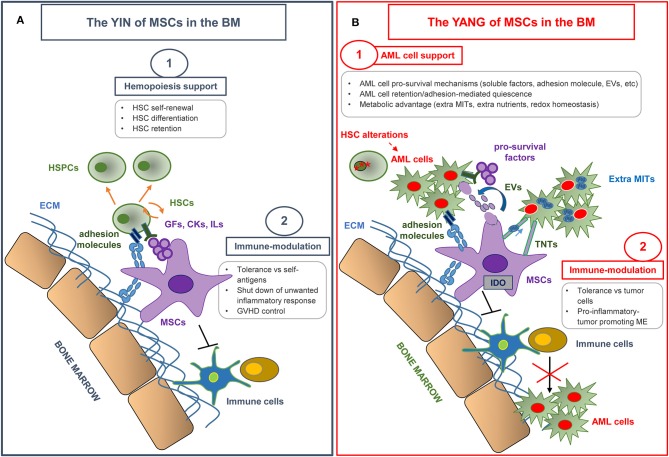
The yin and yang of MSCs in the bone marrow microenvironment. **(A)** MSCs make a substantial contribution to the creation of the hematopoietic *niche*, and play an essential role in normal hematopoiesis by regulating hematopoietic stem cell (HSC) proliferation and differentiation. MSCs also display a unique immune regulation ability by inhibiting the activation, proliferation, and function of both adaptive and innate immune cells. MSC immunomodulatory properties have been extensively used in various clinical settings, in particular in graft vs. host disease (GVHD) control. HSPCs, hematopoietic stem and progenitor cells; ECM, extracellular matrix; GFs, growth factors; CKs, cytokines; ILs, interleukins. **(B)** MSCs have revealed an emerging role in creating a protective and immune-tolerant microenvironment able to support AML survival and to affect therapy response. The most relevant MSC-dependent processes nurturing a leukemia drug-resistant phenotype are outlined. AML, Acute Myeloid Leukemia; EVs, extra vesicles; TNTs, tunneling nanotubes; MITs, Mitochondria; ME, microenvironment.

MSCs represent one of the fundamental components of the hematopoietic niche (9, 10) (Figure 1A). *In vivo*, the niche provides a microenvironment which controls the maintenance, self-renewal, and differentiation of hematopoietic stem cells (HSCs), also regulating the release of mature progeny into the vascular system. In addition, the niche protects HSCs from stimuli which would exhaust the stem-cell reserves (11). MSCs actively contribute to the creation of the HSC niche. Indeed, when transplanted in mice, MSCs have been shown to differentiate into osteoblasts, pericytes, BM stromal cells, osteocytes, and endothelial cells, which all represent functional elements of the niche able to support hematopoiesis (12).

BM-MSCs also favor the engraftment and homing of HSCs in the BM of animals when co-transplanted (13–15). The capacity of MSCs to support hematopoiesis was demonstrated *in vitro* (16) and both direct cell-to-cell contact and release of soluble factors seem to be involved (17). Human MSCs produce a wide variety of cytokines favoring HSC quiescence or self-renewal, i.e., stem cell factor (SCF), stromal cell-derived factor (SDF-1), bone morphogenetic protein 4, transforming growth factor (TGF)-β, leukemia inhibitory factor (LIF), and other cytokines influencing more mature hematopoietic progenitors e.g., granulocyte macrophage colony-stimulating factor (GM-CSF), and granulocyte colony-stimulating factor (G-CSF) (16). MSCs also produce several interleukins (i.e., IL-1, IL-6, IL-7, IL-8, 1L-11, IL-12, IL-14, IL-15). The importance of cell-to-cell contact has been suggested by the demonstration that CD34^+^ cells adhere to the MSC feeder layer, due to the expression of proteins such as cadherins, integrins, vascular cell adhesion molecule, and neural cell adhesion molecule 1. This adhesion is essential to maintain primitive hematopoietic progenitors in culture (18). *In vivo*, different hematopoietic stem and progenitor cell subsets occupy distinct locations in the BM. Analysis of murine BM sections revealed that approximately 85% of HSCs are localized within sinusoidal blood vessels, suggesting the existence of a perivascular niche and its fundamental role in regulating HSC fate (19). On the contrary, <20% of HSCs are located close to the endosteum (within 10 μm) (20, 21). Current data indicate that HSCs localize in the BM according to the stage of differentiation (21), e.g., early lineage-committed progenitors reside preferentially in the endosteal niche (9). Given that the position of HSCs within the niche is critical, it is fundamental to define the features of stromal cells associated with the different locations. However, univocal surface markers for MSC identification and classification *in vivo* have not yet been found. Most of the available data were obtained in the mouse model. In 2006, Sugiyama et al. defined mesenchymal progenitors as the cells expressing an elevated level of the HSC maintenance protein, CXCL12 (SDF-1), the so called CXCL12-abundant reticular (CAR) cells. CAR cells are quite abundant in BM and are found in close contact with putative HSCs, in proximity to sinusoidal vessels and to endosteum (22). In 2007, another population of subendothelial osteoprogenitor cells was identified close to sinusoids. These cells were positive for the melanoma cell adhesion molecule (CD146^+^) and show MSC activity, i.e., the ability to transfer the hematopoietic microenvironment upon heterotopic transplantation (23). Finally, Mendez-Ferrer et al. identified a putative mesenchymal population, expressing Nestin (Nestin^+^ MSCs), a protein typical of neural cells. Nestin^+^ MSCs show CFU-F content, multilineage differentiation, and self-renewal ability. Nestin^+^ MSCs are closely associated with HSCs and reside in the perivascular area, and with a lower frequency in the immediate vicinity of the endosteum. *In vivo*, selective depletion of Nestin^+^ MSCs reduces HSC number and BM homing of transplanted HSCs (24). Nestin^+^ MSCs are less abundant than CAR cells and express CXCL12 as well. Thus, Nestin^+^ MSCs may hold a more primitive phenotype compared to CAR cells (25). To date, is still under debate whether the different niche populations described in mice and therefore the spatial relationship between the different niche cells and HSCs are preserved in humans. As markers for human MSC identification, nerve growth factor receptor (CD271) and CD146 have been indicated. Indeed, CD271^+^ cells are able to support hematopoiesis and to form CFU-Fs with tri-lineage differentiation potential *in vitro* (26), and CD146 defines a subset of CD271^+^ cell populations with different locations: endosteal cells (CD146^−^) or perivascular cells (CD146^+^) (27), which express HSC maintenance genes (28, 29). These cells also express other markers such as CD105 and CD90 (30, 31).

### MSC Immunomodulatory Properties *in vitro*

MSCs are considered to be hypoimmunogenic, because they express low levels of human leukocyte antigen class II (HLA-II) and co-stimulatory molecules including CD40, B7, CD80, and CD86, and they do not stimulate alloreactive T lymphocyte responses *in vitro* (32, 33). Moreover, a well-described characteristic of MSCs is their immune regulation ability, which influences both adaptive and innate immunity (34) (Figure 1A). The immunomodulatory effect of MSCs relies on immunological conditions in the local microenvironment, where inflammatory surroundings influence MSC behavior. In particular, interferon (IFN)-γ and tumor necrosis factor (TNF)-α play a key role in inducing the immunosuppressive ability of MSCs and in creating an immunosuppressive microenvironment. This effect is desirable to induce self-tolerance and to control a potentially harmful inflammatory response, but, as explained in detail below, it is deleterious when it suppresses the response against cancer cells.

MSCs influence the activity and functions of various immune cells both via soluble factors and cell-to-cell contact mechanisms. *In vitro*, autologous or allogeneic MSCs are able to inhibit T-cell proliferation induced by distinct stimuli, including mitogens, alloantigens, and CD3/CD28 mediated activation (34–37). Interestingly, MSCs do not induce T-cell apoptosis, but favor their survival in a quiescent state, promoting the arrest of T cells in the G0/G1 phase of the cell cycle (38, 39). The inhibitory effect of MSCs on T cells also requires MSC–T-cell contact (40). The activation of toll-like receptor (TLR)-3 and TLR-4, expressed on human BM-derived MSCs (41), induces pro-inflammatory signals and hampers the negative activity of MSCs on T-cell proliferation (42). In turn, MSCs could be polarized, depending on TLR activation, into different phenotypes: a pro-inflammatory MSC1 phenotype when TLR4-primed, and an immunosuppressive MSC2 phenotype following TLR3 activation (43). Furthermore, MSCs affect T-cell cytokine production: murine MSCs induce a decrease of IFN-γ production by T helper 1 (Th1) cells *in vivo* (44) and human MSCs increase IL-4 production by Th2 cells *in vitro* (34), skewing the phenotype from a pro-inflammatory to an anti-inflammatory state (11). As discussed in detail below, the suppressive activity of MSCs is in part mediated by indoleamine 2,3-dioxygenase (IDO)-1 expression and activity, stimulated in turn by IFN-γ/TNF-α producing activated T cells (45, 46). Additionally, MSCs inhibit naïve Cytotoxic T lymphocyte (CTL)-mediated lysis, through the release of soluble factors. MSCs are not lysed by CTLs, suggesting the existence of a mechanism which allows MSCs to escape recognition by CTLs (47).

MSCs are able to induce regulatory T cells (Tregs) (34). In particular, MSC-exposed Tregs have increased immunosuppressive activity, compared to Tregs not pre-cultured with MSCs. This effect is potentially due to the activation of programmed cell death 1 receptor (PD)-1 on Tregs and IL-10 production in MSC/Treg co-culture system (48). In addition, prostaglandin E2 (PGE2), TGF-β, and HLA-G5 expression in MSCs, as well as contact-dependent mechanisms, are responsible for MSC-mediated Treg induction (49, 50). MSCs can indirectly induce Tregs by inducing the production of IL-10 in dendritic cells (DCs), which in turn induces the Treg generation *in vitro* (34).

It has been shown that MSCs may block DC differentiation from peripheral or cord blood-derived precursors (51). Likewise, MSCs prevent the typical expression of surface markers such as CD80, CD86, and HLA-DR during DC maturation (52). MSCs affect mature DC function, inducing a decreased expression of major histocompatibility complex class II and other proteins and a decreased IL-2 production, which in turn alter antigen presenting cell (APC) activity of DCs (53). In addition, MSCs reduce TNF-α secretion by DCs and hence reduce their pro-inflammatory activity (34). The negative regulation of DCs, mediated by MSCs, is exerted through several mechanisms, including MSC secretions of IL-6 (54) and PGE2 (55). Recently, MSC-derived extracellular vesicles (EVs) have been reported to reduce the maturation and function of DCs (56).

MSCs are involved in the regulation of natural killer (NK) cells and macrophages. MSCs are able to block resting NK cell proliferation and cytotoxicity, whereas MSC effects on NK activated cells are less evident (57). In addition, MSCs inhibit cytolysis mediated by NK cells and their IFN-γ production, through HLA-G5 secretion (49). It is noteworthy that IL-2 activated NKs, contrary to freshly isolated NKs, are able to lyse autologous and allogeneic MSCs (57). Finally, murine MSCs promote macrophage M2 polarization *in vitro* through activation of signal transducer and activator of transcription (STAT)-3 and inhibition of nuclear factor kappa-light-chain-enhancer of activated B cells (NF-κB) (58). Human MSCs program macrophage plasticity, favoring the M2 phenotype through PGE2 release and altering macrophage metabolic status (59). In turn, M2 macrophages, contrary to their M1 counterpart, are able to induce MSC osteoblast differentiation, suggesting their particular role in regulating bone homeostasis (60). Interestingly, MSCs can recruit macrophages/monocytes into the inflammation site through the release of paracrine factors (61).

### MSC Immunomodulatory Properties *in vivo*

Given their immunomodulatory properties, MSCs have been extensively used in the clinical setting in recent years. In some areas, such as chronic degenerative disorders, genetic diseases, and solid organ transplantation, treatment with MSCs is in the early phases of development. In other fields, such as steroid refractory acute GVHD (sr-aGVHD), MSCs have already produced significant results, and might be considered as a treatment option (Figure 1A). Immunomodulation through MSCs has some properties which make it particularly useful for the treatment of sr-aGVHD. In particular, it does not require donor-recipient matching, it is non-antigen specific and, last but not least, it is dependent on exposure to inflammation (“site specific”) (62). Accordingly, MSCs have been widely tested as a salvage option to treat sr-aGVHD, and convincingly shown their effectiveness by improving overall survival (OS) of responding patients (63, 64). Despite encouraging results reported within the last 10 years, there is still an urgent requirement to find well-defined factors that can be used to predict treatment outcome at an early stage, in order to identify those patients more likely to respond and the most effective administration regimen. A better understanding of these matters would considerably optimize MSC treatment and help doctors to clearly establish the possible role of MSCs in the therapeutic management of GVHD (65). An exhaustive discussion of the clinical trials conducted up to now is not an objective of this review. The results of the most relevant clinical studies with MSCs for sr-aGVHD, conducted in the last 10 years, are reported in Table 1.

**Table 1:** MSCs for GVHD.

**Study**	**N. of patients**	**Cell dose (10^**6**^/Kg)**	**Response rate (%)**	**Overall survival (%)**
Resnick et al. (66)	50	1.1		25 (80 in responders)
von Dalowski et al. (64)	58	0.99	47	19 at 1 year
Introna et al. (67)	40	1.5 (3 doses)	67.5	50 at 1 year
Sánchez-Guijo et al. (68)	25	1 4 sequential doses (all 2)	71	N/R
Servais et al. (69)	33 (2 cohorts)	1–2 vs. 3–4	21 vs. 30	0 vs. 48
Bader et al. (70)	69 (51 children + 18 adults)	1–2	30 CR−9 PR−16 NR	75 (children) 61 (adults)

## The Yang of MSCs: MSC Role in AML Onset and Progression

AML and other myeloid malignancies have for a long time been considered exclusively driven by leukemic cell-intrinsic mechanisms, due to the discovery of critical driver mutations in HSCs (71, 72). Recently, the role of the microenvironment and BM stromal cells, including MSCs, gained attention as critical contributors to AML pathogenesis, persistence, and recurrence. Recent findings suggest that alterations, which first occur in the BM niche, can directly drive the dysfunction of HSCs, favoring leukemia initiation and progression in a niche-driven model of malignant transformation. Moreover, growing evidence highlights the ability of leukemic cells to profit from physiological signals and to shape BM niche cells (HSC-driven model), in order to create a self-reinforcing and more favorable niche, supporting malignant cells at the expense of normal hematopoiesis.

### Niche-Driven Mechanisms of Leukemogenesis

The BM microenvironment exerts not only a bystander effect but also plays an active role in myeloid transformation. The genetic manipulation of specific stromal cell subsets is able to drive leukemogenesis in mice. In a first study, the targeted deletion of the Dicer I, ribonuclease III (*Dicer I*), in a well-defined population of mesenchymal progenitors recapitulates features of human myelodysplastic syndrome (MDS), including the propensity to develop into AML (73). The loss of *Dicer I* results in a decrease of the SBDS ribosome maturation factor (*Sbds*) expression in mesenchymal/osteoprogenitor cells. *SBDS* encodes for a protein involved in ribosomal maturation and its mutation has been identified in Shwachman-Diamond syndrome (SDS), characterized by BM failure with a high risk of developing AML (73). MSCs isolated from MDS patients show a lower expression of DICER I and SBDS (74). Furthermore, mesenchymal progenitors devoid of the *Sbds* gene show increased secretion of damage associated molecular pattern (DAMP) molecules, S100A8 and S100A9, leading to mitochondria dysfunction and genotoxic stress in HSCs. A correlation was also found between the expression of the two DAMP molecules in MSCs isolated from MDS patients and the increased risk of these patients of developing AML (75).

The role of MSC-derived osteoprogenitors in AML has recently been highlighted. The activating mutation in the β-catenin gene in osteoprogenitor cells causes the activation of Notch signaling in HSCs, and impairs myeloid and lymphoid differentiation leading to the development of AML in mice. Interestingly, 38% of MDS and AML patients show an increase of β-catenin signaling in osteoblasts and of NOTCH signaling in HSCs (76, 77). Finally, activating mutations of the Tyrosine phosphatase SHP-2 (encoded by *Ptpn11* gene) in MSCs and osteoprogenitors, already found in Noonan syndrome and associated with an increased risk of leukemic transformation, induce juvenile myelomonocytic leukemia-like myeloproliferative neoplasm in mice (78).

A niche-driven mechanism of leukemia in humans is supported by the phenomenon of a donor cell-derived hematopoietic neoplasm, reported for AML, MDS, T-cell lymphoma, and chronic myeloid leukemia (CML). Indeed, in rare cases, donor-derived leukemia occurring in patients receiving BM transplantation may be completely different from the original leukemic clone. In the host, pre-existing BM niche alterations could initiate leukemogenesis in engrafted cells of donor origin (79, 80). In addition, a perturbation of those pathways, which we mentioned before as able to drive a niche-induced transformation in mice (i.e., Dicer1, sbds, S100A8/9, β-catenin), has been described in patients (73–76). *Ex vivo* expanded MSCs isolated from MDS and AML patients show several alterations, e.g., chromosomal aberrations in MSCs derived from MDS and AML patients have been reported in 30–70% of the samples. Surprisingly, in some patients, the cytogenetic abnormalities identified in MSCs are different from those detected in hematopoietic cells, isolated from the same patients (81, 82). Chromosomal and genetic alterations of MSCs have been correlated to specific gene-expression programs and disease subtypes, suggesting that the genetic susceptibility of MSCs can play an active role in the progression of MDS and AML (83). However, the functional meanings of these alterations are still under debate and conflicting results have been published. Several studies reported normal functionality including differentiation capacity, ability to support hematopoiesis *in vitro*, immunophenotype, expression of adhesion and extracellular matrix proteins (84–86). On the contrary, other studies revealed functional alterations of MSCs, including growth deficiency and altered osteogenic differentiation ability, and reduced capacity to support hematopoietic cells (83, 86–91). The secretome of MSCs is also altered in the leukemic BM (87, 92, 93). In particular, it has been demonstrated that a subset of MSCs shows an increased expression of pro-inflammatory molecules e.g., C-C motif chemokine ligand 3 (CCL3), TNF, IL-8, IL-6, DAMPs, and a reduced expression of factors essential for HSC maintenance and differentiation e.g., CXCL12, KIT Ligand, and angiopoietin 1 (28, 61, 75).

### HSC-Driven Mechanisms of Leukemogenesis Exploiting the BM Microenvironment

Malignant HSCs can influence the composition and the functional status of the BM microenvironment. Medyouf et al. found that primary human MDS cells could instruct MSCs, isolated from healthy donors (HDs), toward an MDS-like behavior *in vitro* (94). Furthermore, in a patient-derived xenograft model, MDS cells need their disease-associated MSCs to propagate the disease *in vivo*, suggesting that both hematopoietic and stromal cells have a role in MDS (94). Recently, the remodeling of the niche, induced by AML cells, has been characterized. Similarly to MDS, healthy MSCs cultured in AML cell-conditioned medium showed a reduced osteogenic differentiation and proliferation ability (87). Among the mechanisms driving the remodeling of the niche, inflammation, a hallmark of cancer, seems to play a role. The ability to induce inflammation could potentially reflect specific “physiological” immune functions, still present in malignant cells (95). In an AML mouse model, it was observed that CCL3 produced by AML cells inhibits osteoblastic cells and bone demineralization. Interestingly, CCL3 mRNA is detected also in malignant cells isolated from AML patients (96).

AML cells are able to modify the transcriptome of MSCs. Indeed, leukemic stem cells (LSCs), unlike normal CD34^+^ cells, modulate in MSCs the expression of cell-cycle genes, cytokine-related genes, and other genes, like *CXCL12* and *JAG-1*, involved in cell-to-cell cross-talk (91).

Additionally, malignant cells can shape the niche through the release of EVs. EVs (e.g., exosomes and microvesicles) are new mediators in the intercellular communication network in various physiological and pathophysiological frameworks. EVs are produced starting from different cell types and perform different functions depending on their origins and cargoes. Indeed, EVs contain proteins, lipids, and nucleic acid, representing potential signal molecules to deliver to specific target cells (97, 98). Both primary AML cells and AML cell lines release EVs, carrying several coding and non-coding RNAs, relevant to AML pathogenesis. It has been demonstrated that the content of AML cell-derived EVs is able to modify stromal cell functions, regulating cellular pathways including the production of growth factors, metabolism, and immune response, all favoring AML cell survival (99–102). *In vivo*, AML-derived EVs mediate the BM remodeling leading to an increased number of mesenchymal stromal progenitors, osteoblast loss and downregulation of HSC-supporting factors in BM stromal cells. The disruption of EV secretion reduces leukemia progression in mice (103).

Kim and collaborators found that distinct patterns of MSC changes induced by AML cells characterize AML patients and are associated with a heterogeneous clinical course. Stromal remodeling in leukemic BM may potentially serve as a prognostic factor (91). All these mechanisms indicate a fundamental bi-directional interaction among malignant cells and surrounding microenvironment (83) with relevant consequences on AML onset and, as we will explain below, on disease development and outcome.

### Emerging Microenvironment-Driven Mechanisms of Drug Resistance

Despite the introduction of new anti-leukemic agents and approaches, and the high rates of remission after induction therapy, treatment failure, and relapse are still major hurdles in AML. The persistence of AML cells after therapy, resulting in minimal residual disease (MRD), is the main factor responsible for AML relapse. In addition to cell-autonomous mutations (i.e., intrinsic factors), increasing evidence indicates that the BM microenvironment (i.e., extrinsic factors) may contribute to protect LSCs from being killed by chemotherapeutic agents, resulting in MRD persistence (104–107). Indeed, environment-mediated *de novo* drug resistance creates a transient state of malignant cell protection eventually leading to the selection and outgrowth of cells with an increasing level of acquired drug resistance (108). Here, we will describe the most relevant microenvironment-dependent processes underlying protection against chemotherapy, with a particular emphasis on new emerging mechanisms driven by MSCs to nurture a leukemia drug-resistant phenotype (Figure 1B).

#### MSC-Dependent Multi-Drug Resistance Phenotype

The tumor microenvironment can facilitate the establishment of a multi-drug resistance (MDR) phenotype in leukemic cells. Indeed, MDR may arise through different processes, including drug uptake and imbalance in cell survival/death signaling, but especially through the modulation of the adenosine triphosphate-binding cassette (ABC) efflux transporter expression. The ABC transporter regulates the efflux of xenobiotics including chemotherapeutic drugs. In co-culture experiments, a subset of BM-derived stromal cells increased the expression of several ABC transporters in a myeloid leukemia cell line and protection against chemotherapy-induced apoptosis (109, 110). The adhesion of leukemic cells to the BM-microenvironment is also crucial for the persistence of MDR in AML (107).

#### MSC-Dependent Pro-Survival Effect: Soluble Factors and Cell-to-Cell Contact

The ultimate result of a successful cytotoxic treatment is the induction of apoptosis, a genetically determined process of programmed cell death. The BM microenvironment offers protection against cytotoxic agents, allowing the activation of anti-apoptotic signals and leading to enhanced cell survival and resistance to therapy. The activation of signals which inhibit apoptosis is correlated with a poor response to chemotherapy in AML (111–115).

BM stromal cells play a key role in the activation of pro-survival mechanisms. Earlier studies showed that stromal cells protect leukemic cells from apoptosis induced spontaneously, by serum deprivation or by drugs (116–119). It is still unclear whether the pro-survival effect of stromal cells on AML is either due to direct contact (116, 117, 120) or to soluble factors secreted from stromal cells (118). Regarding the latter, compounds able to reduce IL-6 production by BM stromal cells are cytotoxic for AML cells, but not for BM stromal cells themselves (121). Other growth factors affecting BM-mediated resistance to chemotherapy include TGF-β, basic fibroblast growth factor, and vascular endothelial growth factor (122, 123).

Both soluble factors and adhesion molecules activate pro-survival pathways and often interact with each other. SDF-1/CXCL-12, constitutively expressed by both MSCs and MSC-derived osteoblasts, could stimulate malignant cell survival (124). The SDF-1 receptor, CXCR4 is expressed by normal and malignant hematopoietic cells (125). Anti-CXCR4 or a CXCR4 antagonist decrease AML cell survival *in vitro* (126). Although SDF-1 has not been directly implicated in drug-resistance, it could contribute by an indirect mechanism. Indeed, SDF-1/CXCR4 axis inhibition could override BM stromal cell protection to drug-induced apoptosis in AML (126, 127). CXCR4 antagonists dramatically reduce AML load in mice previously engrafted with primary human AML without influencing the engraftment of normal hematopoietic progenitors (128), probably because tumor cells express higher levels of CXCR4 than normal cells (125). Furthermore, SDF-1 enhances integrin α4β1 (VLA)-4-mediated adhesion of tumor cells to extracellular matrix components (ECM), i.e., fibronectin and collagen, in the BM. Malignant hematopoietic cells adherent to ECM via VLA-4 showed an adhesion-mediated resistance to distinct types of chemotoxic agents (129). VLA-4-mediated adhesion promotes MRD in a mouse model of AML following cytarabine treatment. Anti-VLA4 antibodies reduce MRD and increase mice survival (107). Integrin ligation triggers activation of pro-survival pathways. Integrin-linked kinase interacts with β integrins and activates PI3K/AKT-mediated pro-survival signaling in leukemia cells (120).

Whatever the upstream mechanism, the activation of pro-survival pathways results in the involvement of common targets. BCL2, a critical pro-survival factor, was significantly upregulated in leukemic cells in co-cultures with stromal cells (118, 130). In pre-clinical models, it has been demonstrated that the efficacy of BCL2 inhibitor venetoclax *in vitro* was attenuated by cytokines (i.e., soluble factors) produced by stromal cells (131, 132). Mechanistically, cytokines activate Janus kinase (JAK)/STAT signaling and decrease the expression of BCL-2 relative to the other BCL2 family members (i.e., BCLXL, MCL1, and BFL1). Accordingly, navitoclax, possibly targeting both BCL2 and BCLX, retains its cytotoxic activity also in the presence of the stroma.

As an alternative stroma-mediated pro-survival mechanism, it has been suggested that the microenvironment may contain factors (e.g., adhesion molecules) that can induce malignant cell quiescence contributing to protect them from the drugs targeting rapidly dividing cells. Homing to the niche is crucial for AML LSCs to maintain their stem cell properties, including quiescence. The adhesion molecule CD44, the receptor for hyaluronan, osteopontin, and other ECM molecules, is important for homing and engraftment of AML LSC in murine models. An anti-CD44 antibody inhibits AML LSC engraftment and alters their stem cell fate (133). While BM stromal cells exert their protective effect within the tumor microenvironment, it is likely that away from the niche this protection is no longer active. The mobilization of leukemic cells in the peripheral blood where they exit from dormancy and become subjected to the cytotoxicity of chemotherapeutic drugs could be considered a promising cell killing strategy. Disruption of CXCL-12/CXCR4 interactions with CXCR4 inhibitors efficiently blocks LSC homing to the BM niche and likely sensitizes leukemic cells to chemotherapy (125–128). Based on their ability to mobilize leukemia cells out of protective BM niches, different molecules, including CXCR4 antagonists, have been explored in combination with cytotoxic drugs (i.e., venetoclax).

Conversely, a pro-apoptotic stroma-dependent effect was observed in JAK2-inhibitor treatment (ruxolitinib). In this case, stroma-produced cytokines, such as G-CSF and GM-CSF increased STAT-5 phosphorylation, a downstream target of JAKs, potentiating JAK inhibitor efficacy. When combined with venetoclax, the JAK2 inhibitor ruxolitinib demonstrated synergistic killing activity. This result was summarized in a systemic xenograft model of AML (132). Furthermore, AML cells prove more sensitive to drugs targeting rapidly dividing cells, such as taxanes and vinka alkaloids, in the presence of stroma-secreted soluble factors, probably due to their stimulating effect on cell proliferation (86).

At present, clinical studies with molecules directly targeting stroma are still under construction. Given the genetic diversity of AML, and the complexity of the BM architecture, it will probably be necessary to design studies combining drugs with different targets. Theoretically, the major hurdle is still to find targets particular to the malignant cell population, in order to be able to reduce toxicity and preserve the functions and the properties of the healthy microenvironment. In this view, genomic profiling could be useful in identifying possible targets, also within stroma cells (134).

#### MSC-Dependent Pro-Survival Effect: New Mechanisms

In addition to cell-direct contact and soluble factors, the list of mechanisms accounting for MSC-dependent pro-survival factor transfer has progressively expanded.

*EVs* derived from MSCs (MSC-EVs) are able to vicariate MSCs themselves. Like MSCs, MSC-EVs are able to determine a decrease in pro-inflammatory responses including immune cell activation and oxidative stress (135). Moreover, EVs have been found to retain MSC therapeutic activities *in vivo*, including GVHD improvement (136). EV studies revealed a novel mechanism by which MSCs could play a pro-leukemic function. MSC-EVs act as paracrine factors and induce modifications in the recipient cells that could influence and inhibit proliferation of cancer cells *in vitro* and tumor progression *in vivo*, similar to MSCs (137–139). However, an opposite inhibitory effect on cancer was demonstrated in certain systems (137, 140, 141). Accordingly, EVs could have different effects on cancer cells depending on their content or source (i.e., if produced by normal or malignant MSCs). In particular, EVs derived from normal MSCs inhibit tumor cell proliferation *in vitro* and *in vivo* (142). Although the role of MSC-derived exosome signaling in AML has not yet been established, it has been demonstrated that exosomes derived from MSCs isolated from AML patients contained microRNAs able to influence gene regulatory networks in AML cells (143). In this light, the identification of microRNA and/or proteins that can be transferred from MSCs to leukemic cells represents a promising concept for the development of new therapeutic strategies to treat AML.

*Tunneling nanotubes (TNTs)* have been identified as a novel means for cell-to-cell communications (144–146). TNTs are filamentous actin-based structures, able to connect cells and to act as a route to transport organelles, proteins, and signal molecules, etc. (146, 147). TNTs have been characterized in both cell lines and primary AML cells (148). Interestingly, cytarabine significantly decreases TNTs, while Daunorubicin has no effect. The effect of drugs on TNT number could be an indicator of a different cell sensitivity to the microenvironment-mediated protection. By using a fluorescent tracking system, it was recently demonstrated that TNT signaling extends from MSCs toward AML cells; this could represent a route of mitochondria transfer (149) producing stromal-mediated drug resistance (150). These observations suggest that drugs targeting TNTs could potentially contribute to override the MSC-mediated tumor protection against apoptosis and to prevent drug resistance.

#### MSCs and Mitochondrial Metabolism Regulation

Cancer cells consume high levels of glucose and the majority of them prefer glycolysis even in the presence of oxygen (*Warburg effect*). The prevailing concept is that the *Warburg effect* results from irreversible damage to the oxidative capacity of mitochondria in cancer cells. However, recent findings indicate that the Krebs cycle is intact in leukemia cells (151, 152), and utilizes substrates, such as glutamine and fatty acids, to generate intermediates for biosynthetic pathways and to counteract oxidative stress. In the absence of permanent alterations to the oxidative capacity of the cells, mitochondrial uncoupling (i.e., the abrogation of adenosine triphosphate (ATP) synthesis in response to mitochondrial potential) could mimic the *Warburg effect*. It has been demonstrated that in leukemia cells, MSCs increase the expression of uncoupling protein 2 (UCP2), a mitochondrial inner membrane protein that short circuits the electrochemical gradient generated by the mitochondrial respiration chain. Uncoupled mitochondria display a metabolic shift to the oxidation of carbon sources alternative to glucose, supported in part by fatty acid and glutamine metabolism. Uncoupled mitochondria are more resistant to cytotoxic insults, produce less reactive oxygen species (ROS) and block the activation of the intrinsic apoptotic pathway. Not all leukemic cells are uncoupled and exhibit increased aerobic glycolysis after co-cultures with MSCs (153, 154). Contrary to the Warburg hypothesis, LSCs rely on oxidative phosphorylation to generate ATP (155). LSCs, albeit variable from patient to patient and in some cases within the same patient, are considered the real perpetrators for the propagation of AML. Although the effect of MSCs on mitochondrial uncoupling in LSCs has not yet been evaluated, mitochondrial metabolism has progressively emerged as a putative point of vulnerability for LSCs. Indeed, the inhibition of mitochondrial metabolism selectively targets LSCs, which are metabolically inflexible, i.e., they are unable to shift to glycolysis when mitochondrial respiration is inhibited (152).

Cancer cells could use different strategies to acquire metabolites and organelles, including mitochondria, to build up ATP production. Consistently, it has been shown that AML cells present a higher number of mitochondria in comparison with normal HSCs (156, 157). Interestingly, extra-mitochondria derive from MSCs through a process of transfer that has not yet been defined, but which likely involve TNTs and/or endocytosis and requires cell-to-cell contact (150, 158). AML cells, through NADPH oxidase-2 (NOX2) activity, locally increase oxidative stress, pushing MSCs to increase mitochondria production (150). The extra-mitochondria are transferred to AML cells without any side effect on the metabolic health of MSCs through the activation of peroxisome proliferator-activated receptor γ coactivator (PGC)-1α, the master regulator of mitochondrial biogenesis, which is essential for AML-directed mitochondrial transfer (159). Thus, AML cells with extra-functionally active mitochondria might gain a metabolic advantage, and possibly be relevant for chemo-resistance. Most types of chemotherapy exploit mechanisms involving the induction of oxidative stress. Thus, AML cells with the highest content of mitochondria could most likely survive after oxidative chemotherapy. Accordingly, it has been demonstrated that chemotherapy stimulates mitochondrial transfer (158).

Targeting mitochondria transfer could emerge as an intriguing approach to the development of therapeutic regimens able to tackle chemo-resistant metabolically advantaged AML cells. In support of this theory, normal CD34^+^ HSCs are less prone to receive extra-mitochondria, opening a window for a feasible target therapy with limited detrimental effects. In this context, NOX2 and PGC-1α could be interesting targets, as (1) NOX2 knocked-down AML blasts have reduced mitochondrial respiration; (2) the NOX2 inhibitor decreases mitochondrial transfer and AML cell viability, whereas it is ineffective on normal HSCs, and (3) inhibition of PGC-1α in BM-MSCs reduces mitochondria transport (159).

Moreover, NOX2 and PGC-1α activity appear crucial for AML persistence and recurrence *in vivo*. In fact, NOX2 knocked-down AML mice show better OS with respect to the NOX2 wild type AML control (150), and PGC-1α knocked-down BM-MSCs mice showed a reduced tumor volume compared with control mice (159). Last but not least, extra-mitochondria AML equipped cells could acquire additional anti-apoptotic proteins, also from the BCL-2 family, gaining a survival advantage and, possibly, resistance to standard therapy (160). Promising data, coming from the use of venetoclax, an anti-BCL-2, in elderly AML patients, might partly be exploited by venetoclax's effect on mitochondria and oxidative stress of AML cells (161).

#### MSC-Dependent Nurturing Function

Tumor cells are exposed to nutrient and oxygen-poor conditions. Thus, metabolic adaptation to stress condition and to increased nutrient demand is a crucial requirement for tumor cell survival and expansion. Several data suggest that there is a complex network of metabolic interactions involving malignant cells and their neighbors in the tumor microenvironment. In particular, it is becoming increasingly clear that cancer cells could induce stromal cells to produce metabolites and nutrients to feed their metabolism and gain a proliferative advantage. Among the stromal-produced nutrients, glutamine (GLN), albeit a non-essential amino acid, plays a key role in sustaining the metabolism of proliferating cells and regulating redox homeostasis. A variety of human cancer cell lines, including AML, have been shown to be highly dependent on GLN for proliferation and survival. In particular, AML cells are able to utilize GLN as an alternative carbon source for energy production (162). GLN deprivation or inhibition of Glutamine synthetase (GLS) cause cell growth inhibition and induce apoptosis in AML cells (163).

#### MSC-Dependent Regulation of Redox Homeostasis

Besides nurturing function, the BM microenvironment performs a fundamental role in regulating redox homeostasis of leukemia cells by contributing substrates to generate antioxidants. MSCs, by uncoupling mitochondria, contributed to the reduction of ROS and protection of AML cells from redox-induced damage (153, 164). Recent data have indicated that the leukemia microenvironment in the BM is hypoxic (165). Residing in a hypoxic region may contribute to protect leukemia cells from oxidative stress, including the stress generated by chemotherapy. Furthermore, it has been demonstrated that BM stromal cells contribute to the hypoxic adaptation of leukemic cells. BM-MSCs upregulate hypoxia inducible factor 1 subunit α (HIF-1α) under hypoxic conditions through mTOR/AKT pathway activation. HIF-1α is a key regulator of the cell response to hypoxia and, as a transcription factor, controls the expression of genes involved in energy metabolism, angiogenesis, apoptosis, and cell cycle. HIF-1α induces the stabilization and the activation of the CXCL-12/CXCR4 pathway, thus facilitating the recruitment and the retention of leukemic cells within the leukemic niche (166). HIF-1α up-regulation increases, in turn, the expression of the glucose transporter and drives leukemic cells to switch to glycolytic metabolism. This metabolic adaptation directly inhibits the mitochondrial apoptotic pathway (167). Thus, forced expression of HIF-1α makes leukemic cells resistant to chemotherapy, whereas downregulation of HIF-1α or inhibition of the mTOR pathway restore the chemosensitivity of leukemic cells (168).

#### MSC-Dependent Immunosuppression and Inflammatory Pathways

Among the multifaceted functions attributed to MSCs, immune modulation is the most relevant, from a clinical point of view. Selected pathways of tumor evasion from immune-surveillance, which could potentially lead to anti-cancer therapy resistance, can take place in the BM microenvironment. In solid tumors, it is now well-established that immune suppression mediated by the stromal environment contributes to cancer cell growth (169). However, a similar role for MSCs in AML is less clear. This is partially imputable to the poor characterization of the immunosuppressive and anti-inflammatory properties of MSCs derived from AML patients.

As discussed before, MSCs contribute to the immune responses. Many mediators, including IDO1, PGE2, HLA-G5, IL-10, TGF-β, hepatocyte growth factor, heme oxygenase 1 and LIF, have been involved in the immunosuppressive activity of MSCs, at different levels. It is reasonable to assume that none of these factors alone could lead to a significant abrogation of the immune response. Rather, MSC-mediated immunoregulation is the result of the cumulative action displayed by several molecules simultaneously. Among these molecules, IDO1 performs an immunomodulatory activity in different settings, including AML (170). IDO enzymes catalyze the initial step in tryptophan degradation along the kynurenine pathway, which in turn inhibits T cell proliferation and induces T cell death (171). Thus, professional APCs but also MSCs upregulate IDO1 following inflammatory stimuli, mostly IFN-γ (45). MSCs, expressing IDO1, are able to activate Tregs and to induce T-cell differentiation of Tregs, thereby potentiating immunosuppression in the tumor microenvironment (172). Indeed, Tregs have been recognized as essential contributors in microenvironment immunomodulation and ultimately in helping leukemic cells to evade immune surveillance (173, 174). Data obtained in our lab demonstrated that AML cells, but not normal HSCs, expressed IDO1 (175) which mediates immune tolerance (176) and correlates with a poor clinical outcome (177). We further demonstrated that MSCs isolated from MDS and AML patients also up-regulated IDO1 following pro-inflammatory cytokine treatment to a similar extent with respect to MSCs isolated from HDs (our unpublished data), besides showing comparable immune-regulatory functions (86). However, others demonstrated that AML-derived MSCs are more highly immunosuppressive/anti-inflammatory than those derived from HDs (93). Leukemic cells originate and grow in the immunosuppressive Treg-rich BM microenvironment and are thus protected against the active immune response. On the one hand, immune-modulation could also be exacerbated within an inflamed tumor microenvironment. Inflammatory signals are produced, as mentioned before, both by AML cells and MSCs in the leukemic microenvironment. In particular, TNF-α is upregulated in MSCs after co-culture with leukemic cells (178). Thus, TNF-α, indicated as crucial in all steps of hematologic malignancies (179, 180), may contribute to the generation of an inflammatory niche able to favor tumor growth and immune suppression. On the other hand, the production of an anti-inflammatory/immunosuppressive cytokine IL-10 by MSCs has been indicated as a negative prognostic factor in AML patients, suggesting a dual role of inflammation in the immune response (93). Furthermore, the increase in the MSC-secreted inflammatory cytokine IL-6 could have opposite effects on immunosuppression. While IL-6 has been involved in the inhibition of DC differentiation, decreasing the stimulatory effect of DCs on T cells (53, 54), the secretion of IL-6 by MSCs has been shown to promote anti-tumor adaptive immunity by increasing T lymphocyte trafficking in the tumor microenvironment (181).

Finally, the effect of chemotherapy in the leukemic microenvironment partly relies on the induction of inflammatory modifications, including an increase in serum inflammatory cytokines, thus chemotherapy may lead to the upregulation of IDO1 expression on MSCs, exacerbating an immune tolerant environment. Besides IDO1, the induction of inducible nitric oxide synthase by MSCs and the production of nitric oxide (NO) were shown to play a major role in immunosuppression in murine systems (182–184). However, some data have been published supporting a regulatory role of NO production in MSC-induced immune suppression also in hematological malignancies (178).

All together, these changes in the MSC-dependent inflammatory status of the microenvironment can collectively result in immune modulation and could contribute to tumor progression and drug-resistance within the BM niche.

## Conclusions

Until recently, AML research was focused on the identification of HSC-autonomous and disease-specific genetic events leading to malignant transformation. However, the contribution of the BM microenvironment has gained increasing attention, challenging the evidence that AML derives exclusively from cell-intrinsic defects. New studies have demonstrated that primary alterations of stromal cells, including MSCs, are able (e.g., by promoting an inflammatory or genotoxic microenvironment triggering alterations in HSCs) to induce the disease in mice models and in some cases in patients. Moreover, AML cells exploit MSC-dependent pro-survival signals and shape the BM microenvironment, in order to create a permissive/self-reinforcing niche favorable to escape therapy and immune response. All these concepts converge to indicate a fundamental bi-directional interaction between malignant cells and the BM microenvironment both contributing to AML onset and progression. Although the mechanisms underlying this cross-talk are just starting to be unraveled, an increasing body of evidence indicates that, similarly to other malignancies, targeting the AML microenvironment may be helpful at therapeutical level, thus being complementary to conventional treatments. This approach may lead to improved clinical results, especially for those patients, i.e., high-risk AML sufferers, who still have a dismal prognosis and whose management represents an unmet medical need. In this clinical scenario, a better understanding of cell-extrinsic and microenvironmental mechanisms underlying drug resistance constitutes a fundamental step for the design and development of new and potentially more effective therapies.

## Author Contributions

MCi wrote and revised the manuscript, and was the major contributor. GC wrote and revised sections of the manuscript. FL and FM collected the related papers. GV, AC, and MCa participated in the design of the review and helped to draft and revise the manuscript. AI wrote sections of the manuscript, participated in the design of the review, and helped to draft and revise the manuscript. All authors read and approved the final manuscript.

### Conflict of Interest

The authors declare that the research was conducted in the absence of any commercial or financial relationships that could be construed as a potential conflict of interest.
